# COVID-19 Outcome Prediction and Monitoring Solution for Military Hospitals in South Korea: Development and Evaluation of an Application

**DOI:** 10.2196/22131

**Published:** 2020-11-04

**Authors:** JoonNyung Heo, Ji Ae Park, Deokjae Han, Hyung-Jun Kim, Daeun Ahn, Beomman Ha, Woong Seog, Yu Rang Park

**Affiliations:** 1 Armed Forces Medical Command Seongnam Republic of Korea; 2 Department of Biomedical Systems Informatics Yonsei University College of Medicine Seoul Republic of Korea; 3 Department of Internal Medicine The Armed Forces Capitol Hospital Seongnam Republic of Korea; 4 Department of Nursing The Armed Forces Capitol Hospital Seongnam Republic of Korea

**Keywords:** COVID-19, patient management, prediction model, military medicine, proportional hazards models, outcome, prediction, monitoring, app, usability, prediction, modeling

## Abstract

**Background:**

COVID-19 has officially been declared as a pandemic, and the spread of the virus is placing sustained demands on public health systems. There are speculations that the COVID-19 mortality differences between regions are due to the disparities in the availability of medical resources. Therefore, the selection of patients for diagnosis and treatment is essential in this situation. Military personnel are especially at risk for infectious diseases; thus, patient selection with an evidence-based prognostic model is critical for them.

**Objective:**

This study aims to assess the usability of a novel platform used in the military hospitals in Korea to gather data and deploy patient selection solutions for COVID-19.

**Methods:**

The platform’s structure was developed to provide users with prediction results and to use the data to enhance the prediction models. Two applications were developed: a patient’s application and a physician’s application. The primary outcome was requiring an oxygen supplement. The outcome prediction model was developed with patients from four centers. A Cox proportional hazards model was developed. The outcome of the model for the patient’s application was the length of time from the date of hospitalization to the date of the first oxygen supplement use. The demographic characteristics, past history, patient symptoms, social history, and body temperature were considered as risk factors. A usability study with the Post-Study System Usability Questionnaire (PSSUQ) was conducted on the physician’s application on 50 physicians.

**Results:**

The patient’s application and physician’s application were deployed on the web for wider availability. A total of 246 patients from four centers were used to develop the outcome prediction model. A small percentage (n=18, 7.32%) of the patients needed professional care. The variables included in the developed prediction model were age; body temperature; predisease physical status; history of cardiovascular disease; hypertension; visit to a region with an outbreak; and symptoms of chills, feverishness, dyspnea, and lethargy. The overall C statistic was 0.963 (95% CI 0.936-0.99), and the time-dependent area under the receiver operating characteristic curve ranged from 0.976 at day 3 to 0.979 at day 9. The usability of the physician’s application was good, with an overall average of the responses to the PSSUQ being 2.2 (SD 1.1).

**Conclusions:**

The platform introduced in this study enables evidence-based patient selection in an effortless and timely manner, which is critical in the military. With a well-designed user experience and an accurate prediction model, this platform may help save lives and contain the spread of the novel virus, COVID-19.

## Introduction

COVID-19, which is caused by the SARS-CoV-2 virus, has officially been declared a pandemic [1]. Despite the effort, there were still casualties caused by the disease. The mortality rate of the disease varies between countries and even between cities in China [2]. There are speculations that these mortality rate discrepancies between regions are due to the availability of medical resources in that area. For instance, the mortality rate difference between the city of Hubei and other provinces is significant (~3% for Hubei and 0.7% for the rest of China), and plotting the cases per population against mortality rates shows a positive correlation [2].

The spread of the virus is currently placing sustained demands on public health systems [3]. At present, there is an imbalance in the supply and demand of medical supplies, and many efforts are being made to solve this problem [4]. Although it is ideal if the increase in medical supplies could meet the high demand, this seems to be difficult for many underdeveloped countries.

Adjusting demand, such as by selecting the patients for diagnosis and treatment, can also be an option considering that the rates for severe cases are relatively low, with severe cases at 14% and critical cases at 5% based on Chinese reports [5]. Comparably, rates of the asymptomatic cases are surprisingly high, with some reporting up to 75% [6-8]. In this context, the Centers for Disease Control and Prevention (CDC) recommends that not all patients be hospitalized, considering the insufficient medical infrastructure. However, there is no definite guideline regarding patient selection due to insufficient evidence on which patients need professional care.

Military personnel are especially at risk, since the viral spread is the most critical in settings where people are living closely in groups [9,10]. The adenovirus infection is one of the examples of an infectious disease that is easily spread in the military. Additionally, soldiers are frequently relocated worldwide, which may accelerate the spread of the disease. For instance, in 2007, basic military trainees were infected with the adenovirus and were sent to multiple sites throughout the world, causing an outbreak in those military bases [9].

The confusion, urea, respiratory rate, blood pressure, and 65 years or older (CURB-65) score is a widely known scoring system for predicting the outcome of patients with pneumonia [11]. The presence of confusion, blood urea nitrogen, respiratory rate, systolic and diastolic blood pressure, and age are assessed to calculate a score ranging from 0 to 6, with 6 indicating the highest severity. The American Thoracic Society guidelines suggest that patients with CURB-65 scores of less than 2 are at a low risk of death and may be managed as outpatients. In response to the outbreak, this score was used for patients with COVID-19 [12]. However, even though patients with higher CURB-65 scores were more likely to be severe, more than 20% of the patients with lower scores also had poor outcomes, suggesting the need for a new system to guide patient selection.

Previous studies have developed prognostic models to predict mortality risk, hospital stay, and progression to severe state [13-21]. The largest sample size of these studies was 577 patients from China [19]. Ideally, prediction models should be based on a large data set covering multiple nations and races for vast adaptation. Four of these studies presented models (eg, decision tree, nomogram, and scoring rule) that could be used in clinical practice [14,16,17,21].

When a prediction model is being developed and deployed in the real world, multiple factors should be considered. As previously stated, the included samples should be from multiple centers across multiple nations to account for disparities between races. Additionally, the model should provide a practical and irreplaceable value to its users. The objective of the model should be clear and should be able to answer a major question in the clinical field. The model’s ease of use should also be considered. However, these requirements are difficult—if not impossible—to meet in a situation where a novel infection is spreading worldwide.

In this study, we present a platform that provides outcome prediction and status monitoring for patients with COVID-19 that is consistently enhanced with data collected based on the use of the model.

## Methods

### Development of the Platform Structure

The platform structure was designed to primarily serve the users by providing the results of the prediction models and sending the results to the appointed physician. The data sent by the user to acquire the results were collected by the server with the user’s consent. The newly collected data was used for training the models to further enhance its predictability and generalizability, thereby completing the virtuous cycle. There are two applications employed to serve this purpose: (1) a patient’s application and (2) a physician’s application.

The main functionality of the patient’s application is to provide outcome prediction results to the patient with their general information, previously diagnosed diseases, symptoms, and body temperature. Specifically, the purpose of the outcome prediction is to guide patient selection for medical resource allocation. Thus, the main target of the prediction model is to determine whether the patient will need professional care.

**Figure 1:**
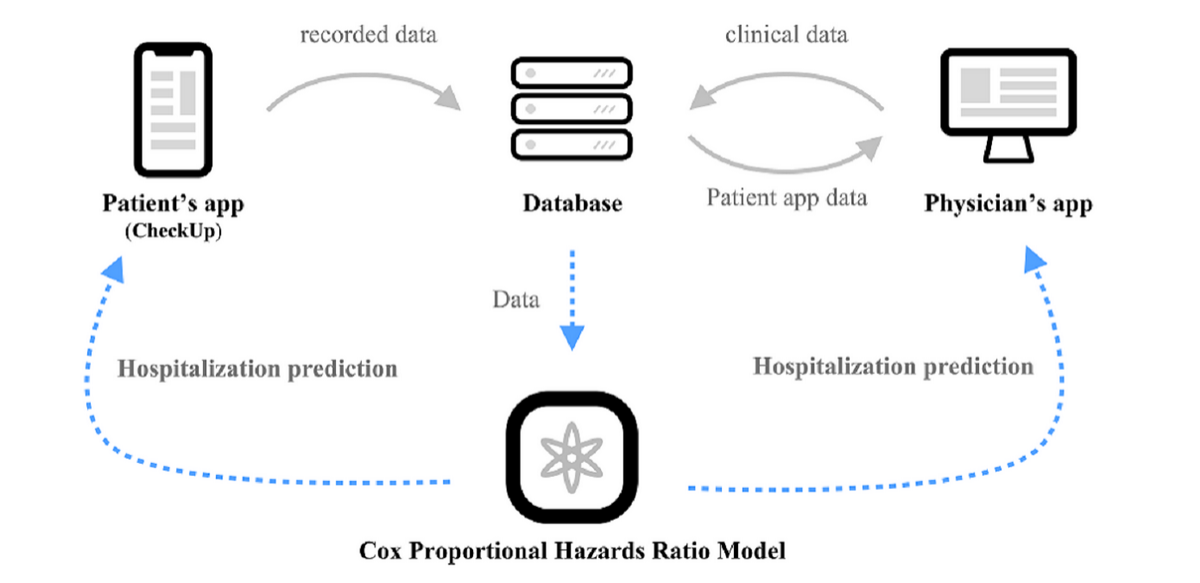
Diagram of the whole structure of the platform.

The application receives the variables that the patient provides to acquire the prediction results, sends them to the central server, and shares them with the appointed physician for remote monitoring. When the patient visits a health care facility, the patient can pair their application with the physician’s application to keep the physician notified of the change in the patient’s symptoms without needing any direct contact. The physician’s application then receives a registration code that can be created from the patient’s application. Upon registering a patient, the health care worker can receive all the records and prediction results from the patient’s application. This process is depicted in Figure 1.

Three physicians reviewed previous publications related to COVID-19 to select the appropriate variables for collection [22-24]. On the basis that this novel virus might have similar features to the pre-existing viruses that cause viral pneumonia, the characteristics of other viral pneumonias were also considered. The selection result was then reviewed by two physicians who are currently directly involved with treating patients with COVID-19 (Multimedia Appendix 1).

### Participants and Data Collection for Outcome Prediction

All patients with COVID-19 admitted to four centers (two military hospitals and two civilian hospitals) in Korea were included in the study. A total of 246 patients were included, and data collection ran from February 6, 2020, to April 2, 2020. There were no exclusion criteria.

The candidate risk factors for the patient’s application included patient demographic characteristics (age, gender, and BMI), past history (asthma, chemotherapy, chronic kidney disease, hypertension, chronic obstructive pulmonary disease, immunosuppressant use, cardiovascular disease, chronic liver disease, and diabetes), patient symptoms (anosmia, rhinorrhea, chest pain, phlegm, chills, physical status, cough, pneumonia, diarrhea, pneumonia, antipyretic, dyspnea, feverish, headache, muscle pain, nausea or vomiting, tired or lethargy, and sore throat), social history (visit to a region with an outbreak, direct contact with a patient with COVID-19, smoking history, household member confirmed with COVID-19, and household member under self-isolation), and body temperature during hospitalization.

The outcome measured in this study was the use of an oxygen supplement monitored up to April 6, 2020—the final date of follow-up. The length of time (days) from the date of hospitalization to the date of first use of an oxygen supplement was considered as our target outcome. Oxygen supplement was chosen as the outcome measure since it may be able to represent the minimal treatment required for hospital admission.

### Statistical Analysis

Descriptive statistics were obtained for all study variables. Continuous data were expressed as mean (SD) values, and categorical data were expressed as proportions.

A survival curve for overall patients was plotted using the Kaplan-Meier method. Using all candidate risk factors, we conducted a univariate analysis by a Cox proportional hazards model to select factors. The factors with *P*<.05 were included in the final model. The body temperature collected during hospitalization was used as the time-dependent variable in the Cox proportion hazards model [25]. By generating time intervals during the day after the date of hospitalization, the average body temperature recorded at each time interval (day) was calculated. That is, the body temperature was considered as a value that changed every day. In case of missing body temperature, the last observation carried forward approach was used. A multivariate Cox proportional hazard model (with time-dependent variables) using selected factors was considered as the prediction model for the patient’s application. The multicollinearity between the factors included in the final model was confirmed through variance inflation factors (VIFs), which quantify the severity of multicollinearity in an ordinary least squares regression analysis. We considered that VIF of 5 or above indicates a multicollinearity problem [26]. Also, additionally, association between factors was examined through phi coefficient between two binary factors, point-biserial correlation between a continuous and a binary factor, and pearson correlation between two continuous factors. To calculate VIF through the ordinary least square regression analysis and to measure correlation between factors, the median body temperature during hospitalization was considered for body temperature with time-varying characteristics.

We calculated the Harrell C statistic to assess the overall predictive accuracy and time-dependent area under the receiver operating characteristic curve (area under the curve [AUC]) [27] to assess the predictive accuracy over the entire follow-up period. The AUC results of the Cox model, which vary depending on the time point to be evaluated, were summarized by days. We also performed the likelihood ratio, Wald, and score tests to measure overall goodness of fit based on the omnibus test of model coefficients. The chi-square goodness-of-fit test proposed by Schoenfeld [28] was used for the proportional hazards. Since body temperature was considered as a time-varying variable updated every day in the model, we did not check the assumption for body temperature. A random subsampling [29] was used to estimate the internal validity of the final Cox proportional hazards model, with 50 repeated samplings dividing the train and test sets into 2:1. The sampling was performed by stratifying according to the occurrence of the outcome event to establish balance in the test and train sets. An average time-dependent AUC based on the 50 repetitions was used as a result of the validation.

The Cox proportional hazards model was considered as a prediction model for the patient’s application, but we additionally constructed a logistic model as an alternative model by using selected factors from the final Cox model. The logistic model was designed to predict oxygen supplement use during hospitalization.

All *P* values were two-sided, and *P*<.05 was considered statistically significant. Statistical analysis was performed using R 3.6.0 (R Foundation for Statistical Computing) [29].

### Evaluation of the Physician’s Application

A usability study was conducted to evaluate the usability of the health care worker’s application wherein participants had to be medical doctors with more than 1 year of clinical practice. A total of 50 physicians were recruited from a public website, and there was no exclusion or selection of the participants. The participants were introduced to the entire platform and briefed about its objectives. After a thorough explanation of the study, a consent form was signed by the participants. They were then instructed to sign up for the service through the physician’s application and then add a prepared sample patient case that was provided. Afterward, participants were required to complete several tasks: review the symptoms that the sample case reported through the patient’s application, review the results from the outcome prediction model of the registered patient, and add additional factitious clinical variables for the patient from the variable input form. The result from the model was presented as the probability of requiring an oxygen supplement, expressed as a number between 0%-100%. The participants were educated on the intended clinical utility of the result from the prediction model to provide supportive information that can be used during patient selection. However, the usefulness of the prediction model could not be assessed since the data were not from a real patient. The participants were instructed to fill the factitious values of the patients’ clinical variables required by the physician’s input form. The study protocol was approved by the institutional review board at the Yonsei University College of Medicine (4-2020-0351).

Following the completion of the tasks, the Post-Study System Usability Questionnaire (PSSUQ) was used to acquire the participants’ responses [30]. The results from the PSSUQ were analyzed using statistical representative indexes for each question.

## Results

### Platform Structure

**Figure 2:**
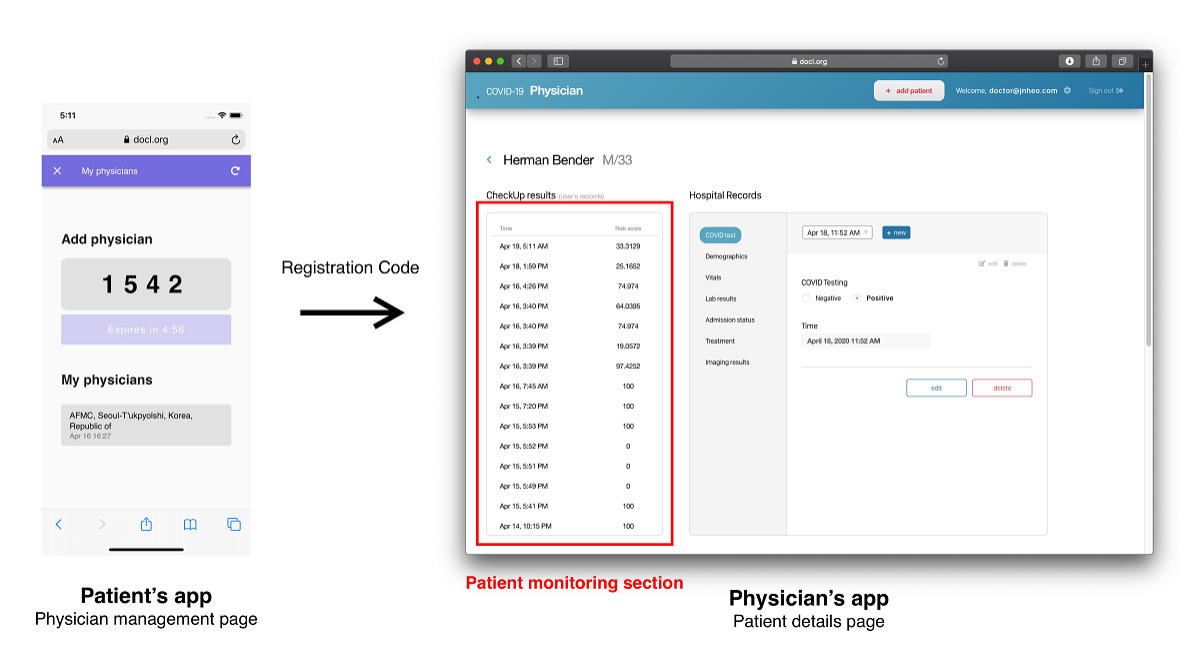
Screenshots showing how patient recorded data is shown in the physician’s app to monitor the patient’s status.

The platform consists of three parts: (1) the patient’s application, (2) the physician’s application, and (3) the model application programming interface. Both the patient’s and the physician’s applications are deployed on the web. The web platform was chosen to be able to enable instant modification upon deployment and maximize the availability on variable devices. Considering the majority of devices the users will be using, the patient’s application is configured to be best viewed on a mobile device and the physician’s application on a desktop. The diagram of the entire structure is illustrated in Figure 2.

This platform is registered in the World Health Organization Digital Atlas for COVID-19 solutions [31].

### Real-World Use of the Platform

A patient is advised to use the patient’s application when they are officially diagnosed with COVID-19. They are also advised to use the application every day and provide information about their daily symptoms and body temperature. If the result from the application shows a high likelihood of requiring hospitalization and the patient is not currently admitted to a hospital, then the patient is advised to contact a nearby health care professional. If there is a previously appointed physician monitoring the patient’s daily symptoms, then the physician can check the daily status of the patient. Using the results of the application, the physician is then able to make a proper clinical decision about whether the patient will need hospitalization. The result that the physician receives is the raw probability calculated from the prediction model. No cut-off was set, since the cut-off will be affected by environmental factors, such as the availability of hospital beds or the regional number of confirmed patients.

### Development of the Outcome Prediction Model

The baseline characteristics of all 246 patients are shown in Multimedia Appendix 2. The mean (SD) of age (year) and BMI (kg/m^2^) were 40.72 (SD 17.10) years and 3.21 (SD 3.28) kg/m^2^, respectively. A total of 167 (67.89%) patients were male. The mean (SD) of the max and median body temperature during follow-up were 37.32 °C (SD 0.56) and 36.75 °C (SD 0.42), respectively. The most common symptom was coughing (95 patients, 41.13%), and 192 (83.12%) patients visited a region with a COVID-19 outbreak.

One patient with missing date of hospitalization among 246 patients was excluded to estimate the survival rate for oxygen supplement. During 2469.32 person-days of follow-up, oxygen supplement was observed in 18 (7.35%) patients. The median (IQR) time from hospitalization to censoring was 17.64 (IQR 14.24-21.70) days. The Kaplan-Meier estimates of the 3-day and 5-day survival rate were 0.947 (95% CI 0.919-0.975) and 0.930 (95% CI 0.898-0.963), respectively.

Table 1 shows results of the univariate and multivariate Cox proportional hazards models. In the univariate analysis, 10 of the candidate risk factors showed results of *P*<.05 (Table 1). It was confirmed that hypertension (hazard ratio [HR] 3.792, 95% CI 1.423-10.109), cardiovascular disease (HR 12.413, 95% CI 4.069-37.869), predisease physical status (HR 1.854, 95% CI 1.24-2.773), dyspnea (HR 13.498, 95% CI 4.527-40.252), feeling feverish (HR 6.282, 95% CI 2.054-19.213), chills (HR 5.727, 95% CI 1.924-17.048), tiredness or lethargy (HR 6.083, 95% CI 1.989-18.607), older age (HR 1.075, 95% CI 1.052-1.098), and higher body temperature (HR 13.147, 95% CI 6.849-25.237) were risk factors for earlier oxygen supplement, while visiting a region with an outbreak (HR 0.291, 95% CI 0.095-0.89) was confirmed as a risk factor that decreased the risk of oxygen supplement.

**Table 1:** Hazard ratio of univariate and multivariate Cox proportional hazards model.

Factors	Univariate model, HR^a^ (95% CI)	Multivariate model^b^	
		HR (95% CI)	*P* value
Age (years)	1.075 (1.052-1.098)	1.035 (0.983-1.089)	.47
Body temperature^b^ (°C)	13.147 (6.849-25.237)	17.431 (2.856-106.371)	.01
Hypertension (yes)	3.793 (1.423-10.109)	0.562 (0.033-9.437)	.81
CVD^c^ (yes)	12.413 (4.069-37.869)	0.217 (0.008-6.111)	.49
Visit to a region of outbreak (yes)	0.291 (0.095-0.89)	3.381 (0.133-86.084)	.28
Physical status	1.854 (1.24-2.773)	4.259 (1.679-10.802)	.007
Dyspnea (yes)	13.498 (4.527-40.252)	3.878 (0.454-33.111)	.43
Feverish (yes)	6.282 (2.054-19.213)	0.321 (0.05-2.073)	.26
Chills (yes)	5.727 (1.924-17.048)	0.905 (0.049-16.705)	.95
Tired/lethargic (yes)	6.083 (1.989-18.607)	1.506 (0.174-13.019)	.62

^a^HR: hazard ratio.

^b^Cox proportional hazards model with time-dependent variable.

^c^CVD: cardiovascular disease.

As a result of multivariate analysis including all 10 factors, body temperature (HR 17.431, 95% CI 2.856-106.371) was found to be the most powerful risk factor for oxygen supplement. Physical status remained statistically significant as a factor (*P*=.007), while the other eight factors were not statistically significant.

The proportional hazards assumption on the factors included in the multivariate model (except for body temperature, since it is considered as a time-varying variable) was checked. Results showed that all factors satisfied the assumption (Multimedia Appendix 3). The multicollinearity and association between the factors included in the multivariate model were not detected (Multimedia Appendices 4 and 5).

**Figure 3:**
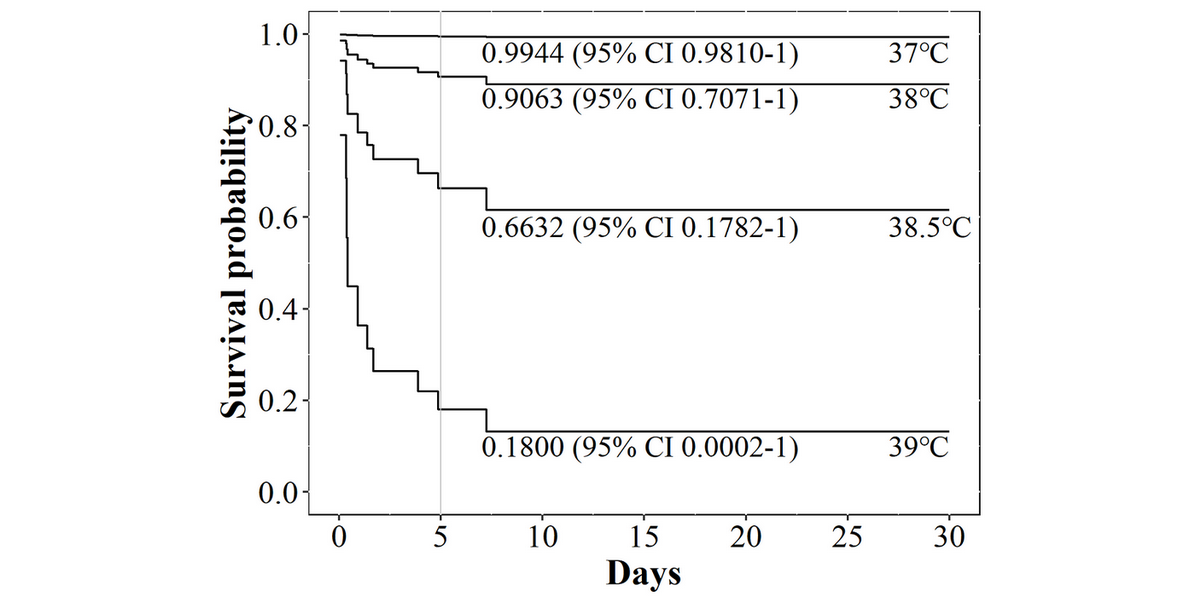
Survival rate according to body temperature in predictive model present in the patient’s application. The 5-day survival rates for each initial body temperature are shown.

To confirm the effects of body temperature in the multivariate model, we considered a case for a fully active man 40 years of age without any past history or symptoms and compared survival rates according to body temperature (Figure 3). In cases of temperatures of 37 °C, 38 °C, 38.5 °C, and 39 °C, the 5-day survival rates were 0.994 (95% CI 0.9810-1), 0.906 (95% CI 0.7071-1), 0.663 (95% CI 0.1782-1), and 0.180 (95% CI 0.0002-1), respectively. At 39 °C, the 5-day survival rate rapidly decreased.

**Figure 4:**
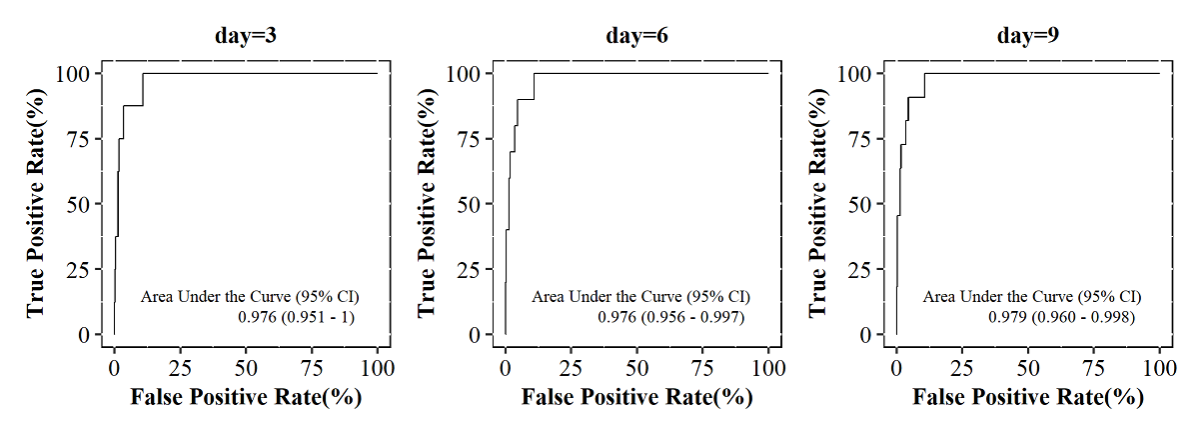
Time-dependent area under the receiver operating characteristic curve at 3, 6, and 9 days from the prediction model present in the patient’s application.

For the multivariate model, the overall C statistic was 0.963 (95% CI 0.936-0.99), and the time-dependent AUC ranged from 0.976 at day 3 to 0.979 at day 9 (Figure 4). Multimedia Appendix 6 shows the time-dependent AUC ranging from day 1 to day 10. The value for each day was above 0.96. The AUC of 8 days or more were the same because the outcome event did not occur after 7.25 days.

As per the result of internal validation for estimating the performance and validity of the multivariate Cox proportional hazards model in Table 1, the average time-dependent AUC ranging from day 1 to day 10 showed a range from a minimum of 0.749 to a maximum of 0.764 (Multimedia Appendix 7).

The results from the model are displayed at the result section of the application when the user has completed a series of questionnaires. The results page shows the 7day survival result based on the received variables.

As results of the multivariate logistic model, body temperature (odds ratio [OR] 19.106, 95% CI 1.587-229.961), physical status (OR 5.145, 95% CI 1.539-17.205), and age (OR 1.099, 95% CI 1.028-1.174) were statistically associated with oxygen supplementation during hospitalization (Multimedia Appendix 8). The *P* values of the likelihood ratio and score tests were less than .001, and the Hosmer and Lemeshow test showed a *P* value of .99. In addition, the AUC was 0.973 (95% CI 0.9413-1; Multimedia Appendix 9).

### Platform Evaluation

The usability study was performed with 50 participants from May 1, 2020, to May 31, 2020. Participants had a mean age of 35.9 years, and 35 (70.0%) were men. All participants were able to complete the given tasks easily. The detailed results are shown in Table 2. The participants were instructed to answer the PSSUQ on a scale of 1-7, with “1” being the most agreeable to the statement. The lower the values of the answers, the better the user experience was. Overall, the participants were satisfied with the app, with the overall average of the responses being 2.2 (SD 1.1). The most disagreeable statement from the questionnaire was the seventh statement (“The system gave error messages that clearly told me how to fix problems”), which had a mean response of 2.7. However, no error was experienced during the study process, thus many of the participants chose “4” as a response, which stood for “neutral.”

**Table 2:** Usability study results (score ranging from 1 to 7, one being “strongly agree”).

Statements	Score, mean (SD)
1. Overall, I am satisfied with how easy it is to use this system.	2.0 (1.0)
2. It was simple to use this system.	2.0 (1.1)
3. I was able to complete the tasks and scenarios quickly using this system.	2.2 (1.1)
4. I felt comfortable using this system.	2.2 (1.2)
5. It was easy to learn to use this system.	2.0 (1.1)
6. I believe I could become productive quickly using this system.	2.0 (1.1)
7. The system gave error messages that clearly told me how to fix problems.	2.7 (1.3)
8. Whenever I made a mistake using the system, I could recover easily and quickly.	2.3 (1.2)
9. The information such as online help, on-screen messages, and other documentation provided with this system was clear.	2.0 (1.0)
10. It was easy to find the information I needed.	2.3 (1.2)
11. The information was effective in helping me complete the tasks and scenarios.	2.1 (1.1)
12. The organization of information on the system screens was clear.	2.0 (1.1)
13. The interface of this system was pleasant.	2.1 (1.2)
14. I liked using the interface of this system.	2.1 (1.2)
15. This system has all the functions and capabilities I expect it to have.	2.4 (1.2)
16. Overall, I am satisfied with this system.	2.1 (1.0)

## Discussion

### Principal Findings

In this study, we developed a platform to provide the users with the most up to date, evidence-based prediction model that can guide them in making decisions on whether to seek professional care or hospitalization, using only the variables provided by the users. The result provided to the users is the probability of the patient requiring oxygen supplement. With this, patients can effectively and accurately monitor themselves on whether they will need hospitalization during self-quarantine. Physicians can use the result from the prediction model for patient selection for hospitalization and risk assessment when needed. The platform collects the data required to calculate the prediction results and uses the data to update the prediction results given directly to the users. The developed Cox model in this study has a high accuracy with an AUC of 0.97 or higher. Our model showed that body temperature was the most important factor for oxygen supplement. A usability study was performed with PSSUQ, which showed that the participants were generally satisfied with the application.

### Timely Patient Selection and the Military

The platform is currently used in military hospitals in Korea. Collection of data and deployment of the outcome prediction model is essential in military settings. If a soldier were to be confirmed with the disease, an exceptionally large number of people would be at risk. In contrast, the patients are likely to be considered low risk, that is, having low probability of requiring professional care, since most of them will be younger and healthier than the general population. This special circumstance, where an explosive spread of the virus is expected but only a few will need special care, will pose a greater importance in the selection of patients for both diagnosis and treatment. A large fraction of the confirmed patients will not be hospitalized, so a close and efficient method for monitoring these patients is necessary. However, the platform was developed considering its use outside of the military when needed. Therefore, patients from two public hospitals were included in the study to address this possibility.

The platform is tailored to fit needs in situations like the COVID-19 pandemic, where data of the novel disease is scarce, and the disease is spreading so fast that traditional clinical trials are not timely enough. Clinical trials are carefully designed to minimize bias and clearly prove a hypothesis. Accuracy and reliability are critical in the medical field, but progressive measures may be required during desperate times. Furthermore, real-world data may be more representative of the patient populations in the clinical field [32]. However, the results derived from this platform should be used in a complementary manner, especially if there is more reliable evidence such as a randomized controlled trial.

The main goal of this platform is to provide the physician with a supportive measure to assess the patient’s risk of requiring professional care. The probability of the patient requiring admission is provided to the physician in the form of a percentage (ie, 0%-100%). The model does not decide if the patient will need hospitalization but provides the risk information of the patient to the physician so a proper decision can be made. Considering the availability of medical resources and the number of patients confirmed with COVID-19, the physician will have to decide which patient will require hospitalization. The prediction model is expected to help the physician with complementary information for the better prioritization of patients.

### Evaluation of the Outcome Prediction Model

The model presented in our study used 246 patients’ data from four centers in Korea to predict whether a patient would need hospitalization during the course of the disease. The Cox proportional hazards ratio model was used to account for the time-dependent variable (ie, body temperature), and the model showed a high predictive accuracy (C statistic: 0.963; AUC at 9 days: 0.979). Ten features were selected in the model: age, body temperature, history of hypertension, cardiovascular disease, visit to a region with an outbreak, predisease physical status, dyspnea, feeling feverish, chills, and tiredness or lethargy. Body temperature showed the highest HR (17.431, 95% CI 2.856-106.371), followed by predisease physical status (HR 4.259, 95% CI 1.679-10.802) and dyspnea (HR 3.898, 95% CI 0.454-33.111; Table 1).

The predictors selected for the model are mostly consistent with previous reports. Although age and comorbidities are already well-established risk factors for grave outcomes [33,34], dyspnea was also found to be associated with disease progression [33]. In our study, the effect of body temperature was the most important predictor. For example, a patient who is a fully active man 40 years of age without any past history or symptoms was highly likely to need an oxygen supplement within 5 days with a 5-day survival rate of 0.18 at 39 °C (Figure 3). Abnormal body temperature is a well-known risk factor for grave prognosis in patients with community-acquired pneumonia [35]. Physical status, which is often expressed in the activities of daily living score, is known to be an independent risk factor for mortality among older adult patients with pneumonia [36,37].

When multivariable prediction models are developed, the number of outcome events compared to the number of predictors, referred to as the events per variable (EPV), affects the accuracy of the model. In general, it is known that a reliable sample size is at least 10 events per predictor (variable) in logistic and Cox models [38,39]. In our model, this rule is not satisfied for the 10 predictors included in the final model. The problem of this low EPV was affected by the accuracy and precision of the coefficients. In addition to the sample size, there is also a possibility that the standard error of the coefficient was overestimated due to the multicollinearity [40], and as a result of the VIF, there was no multicollinearity between predictors (Multimedia Appendix 10). Since overfitting most notably occurs when the number of candidate predictors is large relative to the number of outcome events [41], our model could also be an overfitted model. For this reason, the AUC in the total development sample was about 0.97 (Figure 4), but the result in the internal validation was about 0.75 (Multimedia Appendix 7).

Caution should be employed in the interpretation of several risk factors’ HR in the developed Cox model. We constructed the multivariate Cox model by selecting only statistically significant variables from the univariate model. In the univariate model, the HRs of all risk factors were calculated in the expected direction for outcome risk. However, in the multivariate model, hypertension, cardiovascular disease, feeling feverish, and visits to a region with an outbreak were calculated in a different direction from the result in the univariate model. As these factors have a relatively weak influence compared to body temperature and physical status, and the statistical significance disappeared, caution should be taken in the interpretation of the estimated HRs in contrast to the univariate model.

In the developed Cox proportional hazards model, it is accurate to define the onset of follow-up as the date when the patient was infected with COVID-19. However, it was difficult to accurately estimate the date of infection. Therefore, the onset of follow-up was defined as the date of hospitalization or the date on which the patient first recorded data in the application. The inability to accurately estimate the period between the date when the patient was infected with COVID-19 and the date considered as the onset of follow-up can be considered as a limitation of the model.

### Usability of the Physician’s Application

Usability evaluation was performed for the physician’s application since the use of the application should be effortless to lessen their overwhelming workload due to the COVID-19 pandemic. Additionally, the application will be used in the setting where a physician must monitor a large number of patients; therefore, the usability will have a major impact. The application is simple, presenting a list of associated patients on the first page and showing details of the patient when clicking on the list. Consequently, the use questionnaire showed promising results, with a mean score of the total PSSUQ being 2.2 (SD 1.1). The time taken to complete the given tasks was not recorded, but many participants had completed the tasks under 10 minutes and were surprised by how simple the tasks were. The most disagreeable question was the seventh question, which inquired whether the error message was easy to understand for the user. Since no participant experienced an error, the result from the question was unreliable.

### Other Applications and Outcome Prediction Models for COVID-19

Although not evidence-based, there are some applications that help patients by providing behavioral guidance when they suspect COVID-19 infection. Our initially developed applications, CheckUp Classic and Triage Classic, provided expert opinion-based guidelines in patient selection for testing and patient triage, respectively. These applications received more than 240,000 visits worldwide in a month. Additionally, Apple Inc published a COVID-19 Screening Tool in cooperation with the CDC [42]. A self-triage and self-scheduling tool were also developed based on a well-designed algorithm. This tool is designed to be tethered to the electronic medical records [43].

A few models have been developed to predict the outcome for patients with COVID-19, and a systemic review has been performed [44]. In the review, the authors conclude that all of these models are not useful in the clinical setting. This is primarily due to poor adherence to guidelines and small data size, both of which are unavoidable in a situation where there is not enough time. In a recent editorial, the author emphasizes the importance of sharing data to overcome the limitations of building such a prediction model [45]. However, motive for the researchers to share the hard-earned patient data is weak. Data collection is an additional burden for the already occupied health care providers. There are a few efforts to collect nationwide or worldwide data for patients with COVID-19 [46,47].

COVID-19 Estimated Risk (COVER) was one of the models developed using the common data model [48]. The model comprises nine variables: age, sex, history of cancer, chronic obstructive pulmonary disease, diabetes, heart disease, hypertension, hyperlipidemia, and kidney disease. It was developed based on 6,869,127 patients with influenza or flu-like symptoms. Although the score itself was not originally developed for patients with COVID-19, it was validated with 43,061 patients with COVID-19. The performance, measured with the AUC, was 0.73-0.81 for COVER-H (predicting hospitalization), 0.73-0.91 for COVER-I (predicting intensive care), and 0.82-0.90 for COVER-F (predicting fatality).

### Future Research

Regardless of the accuracy or the representativeness of the data set used for the development of the prediction model, the clinical utility of the platform should be prospectively evaluated. Whether additional information from the prediction model is helpful for the physician or if it is redundant should be further studied. Considering that outcome may vary according to environmental factors, it is questionable if a single prediction model could be generalized worldwide or if there should be separate models for each environment. Currently the platform is not considered for evaluation as an authorized medical device. Extensive prospective research following collection of large data sets to fully represent the target population should be performed.

### Limitations

There are limitations in this study. First, the design of the study does not conform to any previous guidelines for clinical studies. This is due to the difference of scope and objective between traditional clinical studies and this platform. Even so, this study is prone to many types of bias, so the results from this study should be handled with care. Auditing of data is not possible since there is no direct contact with the users, so the completeness or accuracy of the data cannot be ensured. The platform itself is unique, so the study design has never been validated. Considering that the guideline for patient hospitalization is dependent on multiple factors including hospital capacity, governmental guidelines, and patient risk, a single outcome measure cannot be used. Thus, the primary outcome measure—oxygen supplement—is not a definite marker for hospital admission. However, for our data set, all cases were reviewed, and there was no patient that needed hospitalization without receiving oxygen supplement.

Our prediction model was developed from a multicenter data set. However, the model was constructed based on a small data set of a single ethnicity, which can cause selection bias. In addition, since the model has not been built with sufficient data size, there may be a problem in terms of the model’s accuracy. With more data acquisition, we will reinforce our model to expand the population applied to the model and increase the generalizable possibilities by performing external validation for other patients.

### Conclusion

The platform introduced in this study provides evidence-based decision support to guide patient selection in an effortless and timely manner, which is critical in the military. With a well-designed user experience and an accurate prediction model, this platform may help save lives and contain the spread of the novel virus, COVID-19.

## Appendix

Multimedia Appendix 1 Variables collected for the patient’s application.

Multimedia Appendix 2 Overall characteristics of patients at admission.

Multimedia Appendix 3 Chi-square test by using the Schoenfeld residuals for the proportional hazards.

Multimedia Appendix 4 Testing Global Null Hypothesis: Beta=0 of multivariate Cox proportional hazards model.

Multimedia Appendix 5 Correlation matrix between 10 predictor variables included in multivariate Cox proportional hazards model.

Multimedia Appendix 6 Time-dependent area under the receiver operating characteristic curve ranged from 1-day to 10-day of multivariate Cox proportional hazards model.

Multimedia Appendix 7 Average time-dependent area under the receiver operating characteristic curve ranged from 1-day to 10-day using 50 repeated random subsampling to estimate the internal validity.

Multimedia Appendix 8 Odds ratio of multivariate logistic model.

Multimedia Appendix 9 Results of overall model evaluation, goodness of fit, and predictive accuracy from multivariate logistic model.

Multimedia Appendix 10 Results of multicollinearity test with variance inflation factor.

## Abbreviations

AUC: area under the curve
CDC: Centers for Disease Control and Prevention
COVER: COVID-19 Estimated Risk
CURB-65: confusion, urea, respiratory rate, blood pressure, and 65 years or older
EPV: events per variable
HR: hazard ratio
OR: odds ratio
PSSUQ: Post-Study System Usability Questionnaire
VIF: variance inflation factor
