# Supplementary material for: COVID-19 Outcome Prediction and Monitoring Solution for Military Hospitals in South Korea: Development and Evaluation of an Application
Source: J Med Internet Res. 2020 Nov 4;22(11):e22131. doi: 10.2196/22131 (PMC7644266; doi:10.2196/22131)
Supplement: Multimedia Appendix 1 [file jmir_v22i11e22131_app1.docx]

Multimedia Appendix 1. Variables collected for the patient’s application.

|  | Variable name |
| --- | --- |
|  |  |
| **General information** |  |
|  | Birth |
|  | Sex |
|  | Height |
|  | Weight |
| **COVID-19 test** |  |
|  | COVID-19 test result |
| **Previously diagnosed diseases** |  |
|  | Hypertension |
|  | Diabetes |
|  | Cardiovascular disease |
|  | Stroke |
|  | Chronic kidney disease |
|  | Chronic obstructive pulmonary disease |
|  | Asthma |
|  | Chronic liver disease |
|  | Hematologic malignancy |
|  | Transplantation history |
|  | AIDs |
|  | Autoimmune disease |
| **Epidemiologic variables** |  |
|  | Direct contact with COVID-19 patient |
|  | Household member confirmed as COVID-19 positive |
|  | Under self-isolation |
|  | Household member under self-isolation |
| **Others** |  |
|  | GPS coordinates |
|  | Previous physical status |
|  | Antipyretics usage |
|  | Body temperature |
|  | Pregnancy |
|  | Chemotherapy |
|  | Immunosuppressant usage |
|  | Smoking history |
|  | Currently diagnosed pneumonia |
| **Symptoms** |  |
|  | Dyspnea |
|  | Feeling feverish |
|  | Chills |
|  | Myalgia |
|  | Headache |
|  | Fatigue/lethargy |
|  | Sore throat |
|  | Cough |
|  | Sputum |
|  | Rhinorrhea |
|  | Anosmia |
|  | Chest pain |
|  | Diarrhea |
|  | Nausea/vomiting |
