# Supplementary material for: COVID-19 Outcome Prediction and Monitoring Solution for Military Hospitals in South Korea: Development and Evaluation of an Application
Source: J Med Internet Res. 2020 Nov 4;22(11):e22131. doi: 10.2196/22131 (PMC7644266; doi:10.2196/22131)
Supplement: Multimedia Appendix 2 [file jmir_v22i11e22131_app2.docx]

Multimedia Appendix 2. Overall characteristics of patients at admission.

| **Variables** | | | **Total (n = 246)** |
| --- | --- | --- | --- |
|  | | | |
| ***Demographic*** | | | |
| Age, year, mean (SD) | | | 40.72 (17.1) |
| BMI, kg/$m^{2}$, mean (SD) | | | 23.21 (3.28) |
| Gender, male, n (%) | | | 167 (67.89%) |
| *Past history* | | | |
| Asthma, yes, n (%) | | | 2 (0.82%) |
| CKD, yes, n (%) | | | 1 (0.41%) |
| COPD, yes, n (%) | | | 2 (0.82%) |
| CVD, yes, n (%) | | | 8 (3.25%) |
| Diabetes, yes, n (%) | | | 17 (6.91%) |
| Chemotherapy, yes, n (%) | | | 1 (0.41%) |
| Hypertension, yes, n (%) | | | 33 (13.41%) |
| Immunosuppressant usage, yes, n (%) | | | 1 (0.41%) |
| Chronic liver disease, yes, n (%) | | | 6 (2.44%) |
| ***Patient symptoms*** | | | |
| Anosmia, yes, n (%) | | | 11 (4.76%) |
| Chest pain, yes, n (%) | | | 11 (4.76%) |
| Chills, yes, n (%) | | | 32 (13.85%) |
| Cough, yes, n (%) | | | 95 (41.13%) |
| Diarrhea, yes, n (%) | | | 40 (17.32%) |
| Dyspnea, yes, n (%) | | | 21 (9.09%) |
| Feeling feverish, yes, n (%) | | | 52 (22.51%) |
| Headache, yes, n (%) | | | 49 (21.21%) |
| Muscle pain, yes, n (%) | | | 34 (14.72%) |
| Rhinorrhea, yes, n (%) | | | 42 (18.18%) |
| Phlegm, yes, n (%) | | | 71 (30.74%) |
| Physical status, | Fully active, n (%) | | 184 (81.78%) |
|  | Restricted, n (%) | | 35 (15.56%) |
|  | Completely disabled, n (%) | | 6 (2.67%) |
| Pneumonia, yes, n (%) | | | 7 (3.03%) |
| Antipyretic, yes, n (%) | | | 23 (9.96%) |
| Sore throat, yes, n (%) | | | 42 (18.18%) |
| Fatigue/lethargy, yes, n (%) | | | 24 (10.39%) |
| Nausea/vomiting, yes, n (%) | | | 11 (4.76%) |
| ***Social history*** | | | |
| Direct contact with COVID-19 patient, yes, n (%) | | | 67 (29%) |
| Household member confirmed as COVID-19 positive, yes, n (%) | | | 24 (10.39%) |
| Household member under self-isolation, yes, n (%) | | | 25 (10.82%) |
| Visit to a region of outbreak, yes, n (%) | | | 192 (83.12%) |
| Smoking history, | | None, n (%) | 193 (85.78%) |
|  |  | Past, n (%) | 25 (11.11%) |
|  |  | Current, n (%) | 7 (3.11%) |
| ***Others*** | | | |
| Maximum body temperature during follow-up, $℃$ | | | 37.32 (0.56) |
| Maximum body temperature during follow-up, $℃$ | | | 36.75 (0.42) |
