# Supplementary material for: COVID-19 Outcome Prediction and Monitoring Solution for Military Hospitals in South Korea: Development and Evaluation of an Application
Source: J Med Internet Res. 2020 Nov 4;22(11):e22131. doi: 10.2196/22131 (PMC7644266; doi:10.2196/22131)
Supplement: Multimedia Appendix 3 [file jmir_v22i11e22131_app3.docx]

Multimedia Appendix 3. Chi-squared test by using the Schoenfeld residuals for the proportional hazards

| Factor | *P* value |
| --- | --- |
| Age, years | 0.36 |
| Hypertension | 0.18 |
| CVD | 0.24 |
| Visit to a region of outbreak | 0.38 |
| Physical status | 0.93 |
| Dyspnea | 0.71 |
| Feverish | 0.2 |
| Chilling | 0.73 |
| Tired/lethargic | 0.09 |
