# Supplementary material for: COVID-19 Outcome Prediction and Monitoring Solution for Military Hospitals in South Korea: Development and Evaluation of an Application
Source: J Med Internet Res. 2020 Nov 4;22(11):e22131. doi: 10.2196/22131 (PMC7644266; doi:10.2196/22131)
Supplement: Multimedia Appendix 4 [file jmir_v22i11e22131_app4.docx]

Multimedia Appendix 4. Testing Global Null Hypothesis: Beta = 0 of multivariate Cox proportional hazard model.

| Test | Chi-square | DF | Pr>chi-square |
| --- | --- | --- | --- |
| Likelihood Ratio | 49.22 | 10 | <0.001 |
| Score | 145 | 10 | <0.001 |
| Wald | 66.95 | 10 | <0.001 |
