# Supplementary material for: COVID-19 Outcome Prediction and Monitoring Solution for Military Hospitals in South Korea: Development and Evaluation of an Application
Source: J Med Internet Res. 2020 Nov 4;22(11):e22131. doi: 10.2196/22131 (PMC7644266; doi:10.2196/22131)
Supplement: Multimedia Appendix 5 [file jmir_v22i11e22131_app5.docx]

Multimedia Appendix 5. Correlation matrix between 10 predictor variables included in multivariate Cox proportional hazards model.

|  | Hypertension | CVD | Visit to a region of outbreak | Dyspnea | Feverish | Chilling | Tired/lethargic | Age | Physical status | Body temperature |
| --- | --- | --- | --- | --- | --- | --- | --- | --- | --- | --- |
| Hypertension | 1 |  |  |  |  |  |  |  |  |  |
| CVD | 0.199 ^a^ | 1 |  |  |  |  |  |  |  |  |
| Visit to a region of outbreak | -0.1192^a^ | -0.0072 ^a^ | 1 |  |  |  |  |  |  |  |
| Dyspnea | 0.0402 ^a^ | 0.1482 ^a^ | 0.0835 ^a^ | 1 |  |  |  |  |  |  |
| Feverish | 0.0236 ^a^ | 0.1213 ^a^ | -0.2169 ^a^ | 0.1235 ^a^ | 1 |  |  |  |  |  |
| Chilling | 0.0572 ^a^ | 0.0139 ^a^ | -0.0114 ^a^ | 0.3424 ^a^ | 0.5008 ^a^ | 1 |  |  |  |  |
| Tired/lethargic | -0.0302 ^a^ | 0.1263 ^a^ | 0.0604 ^a^ | 0.3724 ^a^ | 0.3746 ^a^ | 0.461 ^a^ | 1 |  |  |  |
| Age | 0.4074^b^ | 0.2925 ^b^ | -0.1466 ^b^ | 0.2049 ^b^ | 0.1103 ^b^ | 0.1613 ^b^ | 0.161 ^b^ | 1 |  |  |
| Physical status | 0.1005 ^b^ | 0.1704 ^b^ | -0.6422 ^b^ | 0.0004 ^b^ | 0.199 ^b^ | -0.0023 ^b^ | -0.0621 ^b^ | 0.2023^c^ | 1 |  |
| Body temperature | -0.0141 ^b^ | 0.1677 ^b^ | 0.3845 ^b^ | 0.2453 ^b^ | 0.1707 ^b^ | 0.3043 ^b^ | 0.2613 ^b^ | 0.0465 ^c^ | -0.2586 ^c^ | 1 |

^a^ Phi coefficient between two dichotomous variables.

^b^ Point-biserial correlation between a continuous and a dichotomous variable.

^c^ Pearson correlation between two continuous variables.
