# Supplementary material for: COVID-19 Outcome Prediction and Monitoring Solution for Military Hospitals in South Korea: Development and Evaluation of an Application
Source: J Med Internet Res. 2020 Nov 4;22(11):e22131. doi: 10.2196/22131 (PMC7644266; doi:10.2196/22131)
Supplement: Multimedia Appendix 6 [file jmir_v22i11e22131_app6.docx]

Multimedia Appendix 6. Time-dependent area under the receiver operating characteristic curve ranged from 1-day to 10-day of multivariate Cox proportional hazard model.

| Time(day) | Area Under the Curve (95% CI) |
| --- | --- |
| 1 | 0.969 (0.937-1) |
| 2 | 0.976 (0.951-1) |
| 3 | 0.976 (0.951-1) |
| 4 | 0.974 (0.951-0.996) |
| 5 | 0.976 (0.956-0.997) |
| 6 | 0.976 (0.956-0.997) |
| 7 | 0.976 (0.956-0.997) |
| 8 | 0.979 (0.96-0.998) |
| 9 | 0.979 (0.96-0.998) |
| 10 | 0.979 (0.96-0.998) |
