# Supplementary material for: COVID-19 Outcome Prediction and Monitoring Solution for Military Hospitals in South Korea: Development and Evaluation of an Application
Source: J Med Internet Res. 2020 Nov 4;22(11):e22131. doi: 10.2196/22131 (PMC7644266; doi:10.2196/22131)
Supplement: Multimedia Appendix 7 [file jmir_v22i11e22131_app7.docx]

Multimedia Appendix 7. Average time-dependent area under the receiver operating characteristic curve ranged from 1-day to 10-day using 50 repeated random sub-sampling to estimate internal validity.

| Time(day) | average Area Under the Curve |
| --- | --- |
| 1 | 0.760 |
| 2 | 0.764 |
| 3 | 0.764 |
| 4 | 0.749 |
| 5 | 0.750 |
| 6 | 0.750 |
| 7 | 0.750 |
| 8 | 0.753 |
| 9 | 0.753 |
| 10 | 0.753 |
