# Supplementary material for: COVID-19 Outcome Prediction and Monitoring Solution for Military Hospitals in South Korea: Development and Evaluation of an Application
Source: J Med Internet Res. 2020 Nov 4;22(11):e22131. doi: 10.2196/22131 (PMC7644266; doi:10.2196/22131)
Supplement: Multimedia Appendix 8 [file jmir_v22i11e22131_app8.docx]

Multimedia Appendix 8. Odds ratio of multivariate logistic model

| Factors | OR (95% CI) | *P* value |
| --- | --- | --- |
| Age, years | 1.099 (1.028, 1.174) | 0.005 |
| Body temperature at admission^a^, $℃$ | 19.106 (1.587, 229.961) | 0.02 |
| Hypertension, yes | 0.565 (0.03, 10.664) | 0.704 |
| CVD, yes | 0.238 (0.004, 14.268) | 0.492 |
| Visit to a region of outbreak, yes | 7.673 (0.224, 262.519) | 0.258 |
| Physical status | 5.145 (1.539, 17.205) | 0.008 |
| Dyspnea, yes | 5.155 (0.601, 44.213) | 0.135 |
| Feverish, yes | 0.327 (0.039, 2.778) | 0.306 |
| Chilling, yes | 2.132 (0.138, 32.814) | 0.587 |
| Tired/lethargic, yes | 2.104 (0.163, 27.095) | 0.568 |

^a^ Average body temperature for the day of admission.
