# Supplementary material for: COVID-19 Outcome Prediction and Monitoring Solution for Military Hospitals in South Korea: Development and Evaluation of an Application
Source: J Med Internet Res. 2020 Nov 4;22(11):e22131. doi: 10.2196/22131 (PMC7644266; doi:10.2196/22131)
Supplement: Multimedia Appendix 9 [file jmir_v22i11e22131_app9.docx]

Multimedia Appendix 9. Results of overall model evaluation, goodness-of-fit and predictive accuracy from multivariate logistic model

| Test | Categories | Chi-square | DF | Pr>chi-square |
| --- | --- | --- | --- | --- |
| Overall model evaluation | Likelihood | 45.34 | 10 | <0.001 |
|  | Score | 55.38 | 10 | <0.001 |
|  | Wald | 17.34 | 10 | 0.067 |
| Goodness-of-fit | Hosmer-Lemeshow | 1.428 | 8 | 0.994 |
| Area under receiver operating characteristic curve | | 0.973 (0.9413 - 1) ^a^ | | |

^a^ 95% CI
