# Supplementary material for: COVID-19 Outcome Prediction and Monitoring Solution for Military Hospitals in South Korea: Development and Evaluation of an Application
Source: J Med Internet Res. 2020 Nov 4;22(11):e22131. doi: 10.2196/22131 (PMC7644266; doi:10.2196/22131)
Supplement: Multimedia Appendix 10 [file jmir_v22i11e22131_app10.docx]

Multimedia Appendix 10. Results of multicollinearity test with variance inflation factor

| Factor | VIF |
| --- | --- |
| Age, years | 1.374 |
| Median body temperature during hospitalization, $℃$ | 1.435 |
| Hypertension | 1.238 |
| CVD | 1.235 |
| Visit to a region of outbreak | 2.006 |
| Physical status | 1.842 |
| Dyspnea | 1.294 |
| Feverish | 1.551 |
| Chilling | 1.705 |
| Tired/lethargic | 1.483 |
